# Paint it black: Efficacy of increased wind turbine rotor blade visibility to reduce avian fatalities

**DOI:** 10.1002/ece3.6592

**Published:** 2020-07-26

**Authors:** Roel May, Torgeir Nygård, Ulla Falkdalen, Jens Åström, Øyvind Hamre, Bård G. Stokke

**Affiliations:** ^1^ Norwegian Institute for Nature Research Norway; ^2^ Lake Ånnsjön Bird Observatory Duved Sweden

**Keywords:** collision fatalities, contrast painting, mitigation measures, motion smear, Smøla wind‐power plant

## Abstract

As wind energy deployment increases and larger wind‐power plants are considered, bird fatalities through collision with moving turbine rotor blades are expected to increase. However, few (cost‐) effective deterrent or mitigation measures have so far been developed to reduce the risk of collision. Provision of “passive” visual cues may enhance the visibility of the rotor blades enabling birds to take evasive action in due time. Laboratory experiments have indicated that painting one of three rotor blades black minimizes motion smear (Hodos 2003, *Minimization of motion smear: Reducing avian collisions with wind turbines*). We tested the hypothesis that painting would increase the visibility of the blades, and that this would reduce fatality rates in situ, at the Smøla wind‐power plant in Norway, using a Before–After–Control–Impact approach employing fatality searches. The annual fatality rate was significantly reduced at the turbines with a painted blade by over 70%, relative to the neighboring control (i.e., unpainted) turbines. The treatment had the largest effect on reduction of raptor fatalities; no white‐tailed eagle carcasses were recorded after painting. Applying contrast painting to the rotor blades significantly reduced the collision risk for a range of birds. Painting the rotor blades at operational turbines was, however, resource demanding given that they had to be painted while in‐place. However, if implemented before construction, this cost will be minimized. It is recommended to repeat this experiment at other sites to ensure that the outcomes are generic at various settings.

## Graphical abstract

Conflicts with birdlife hamper the development of wind energy worldwide. Mitigation measures that reduce the risk of collision by making the rotor blades more conspicuous therefore need to be thoroughly tested. Using a Before–After–Control–Impact approach employing long‐term fatality searches, the annual number of bird carcasses per turbine was significantly reduced at the turbines with a painted blade by over 70%, relative to the neighboring control (i.e., unpainted) turbines.

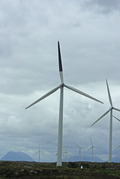

## 1 INTRODUCTION

Climate change concerns (UNFCCC, 2016) have boosted the innovation, development, and application of renewable energy sources worldwide. The global potential for wind‐power generation is enormous (Lu, McElroy, & Kiviluoma, 2009) and regarded by many as the most promising renewable energy source. At the same time, the IPCC Special Report on Renewable Energy (IPCC, 2011) stressed that “environmental and social issues will affect wind energy deployment opportunities.” The construction and operation of wind‐power plants impact wildlife through bird and bat collisions and through habitat and ecosystem modifications, with the nature and magnitude of those impacts being site‐ and species‐specific (Marques et al., 2014; Schuster, Bulling, & Koppel, 2015). As wind energy deployment increases and larger wind‐power plants are considered, existing concerns become more acute and new concerns may arise (Tabassum, Premalatha, Abbasi, & Abbasi, 2014). A goal of the lowest possible environmental costs per kWh from wind energy suggests that wind‐power development in general should focus on sites where there are good wind conditions, adequate infrastructure and where the conflict with environmental issues are acceptable (Langston & Pullan, 2003; OSPAR Commission, 2008). Technological innovation (R&D), together with proactive efforts to mitigate environmental concerns (IPCC, 2011), is expected to lead to further economic and environmental cost reductions for wind energy.

Although comprehensive consent procedures exist to ensure that the best sites are chosen for wind energy production to avoid environmental impacts (Gartman, Wichmann, Bulling, Elena Huesca‐Perez, & Koppel, 2014; Thygesen & Agarwal, 2014), the fast rate of wind‐power development entails that the potential for conflicts increases. Given the fast rate of development, it will become a challenge to verify negative impacts on birdlife and construe ways to minimize these (Langston & Pullan, 2003). Wind turbine‐induced bird mortality is prevalent due to physical collisions with turbines, and so far, few effective deterrent or mitigation measures have been developed to reduce the risk of collisions (Gartman, Bulling, Dahmen, Geißler, & Köppel, 2016b; Marques et al., 2014; May, Reitan, Bevanger, Lorentsen, & Nygard, 2015). Selective shutdown along a migratory flyway for soaring birds (Tomé et al., 2011) and tilling the soil around the tower base (Pescador, Gomez Ramirez, & Peris, 2019) has been found effective in reducing collision rates. However, both measures come at a cost; either through a loss of revenue or annually repeated habitat management, respectively. Development of practical and functional measures to reduce bird mortality related to offshore and onshore wind energy production, that can be industrialized and implemented without delays, is therefore paramount to avoid delay in consenting processes and to streamline the construction and operation phase while conserving species at these sites (May, 2017). Effective measures that reduce the level of avian conflicts may in addition enable development of wind power at new sites and at sites previously declared having too high conflict levels, and improve the utilization of wind resources at specific sites without increasing the conflict levels (May, 2017).

May et al. (2015) provide an overview of the state of the art with regard to postconstruction mitigating measures to reduce bird mortality due to collisions with wind turbines and evaluate their efficacy from an avian sensory, aerodynamic and cognitive perspective. Their main conclusion was that there are few existing tools or measures with a documented effect on bird collisions on the market today. At the same time, there is an increasing demand for such measures, both from the consenting and environmental authorities, nongovernmental organizations, the public, and wind energy developers (Voigt, Straka, & Fritze, 2019). The main reason why mortality‐reducing tools are not readily implemented is due to the challenges concerning the in situ documentation of the effectiveness of such tools (Gartman, Bulling, Dahmen, Geißler, & Köppel, 2016a; Gartman et al., 2016b).

**Figure 1:**
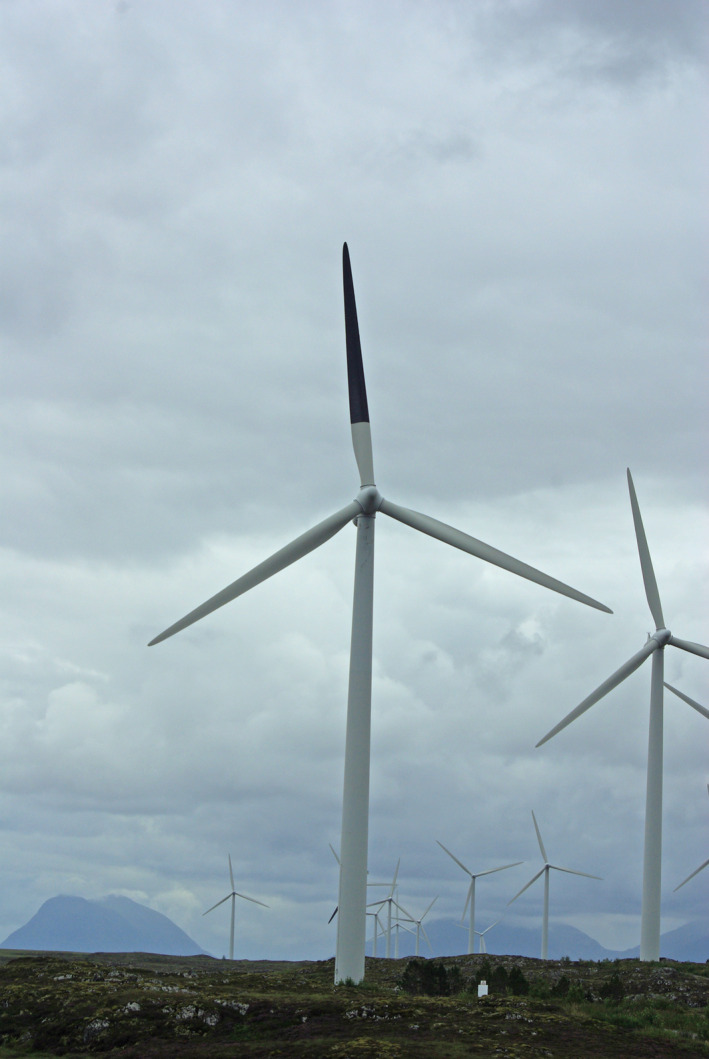
Wind turbine in the Smøla wind‐power plant with painted rotor blade

The majority of bird fatalities at wind‐power plants occur through collision with moving turbine rotor blades. The visual acuity and temporal resolution of the avian eye enable birds to avoid obstacles and to chase prey in full flight and in dim lighting conditions (Jones, Pierce, & Ward, 2007). Relative to humans, birds have a narrow binocular frontal field of view and likely use their monocular and high‐resolution lateral fields of view for detecting predators, conspecifics, and prey (Martin, 2011). Within an assumed open airspace, birds may therefore not always perceive obstructions ahead thereby enhancing the risk of collision. To reduce collision susceptibility, provision of “passive” visual cues may enhance the visibility of the rotor blades enabling birds to take evasive action in due time. Experimental laboratory studies have indicated that painting one of the rotor blades black may help to decrease motion smear (Hodos, 2003). He tested the potential effect of seven blade patterns (striped, staggers, whole black), as well as colored blades, on the retinal‐image velocity (as a measure for the relative visibility above blank rotor blades) in American kestrels (*Falco sparverius*) in a laboratory setting. Hodos (2003) recommended further field tests with a single‐blade, solid black pattern to determine its efficacy in reducing fatalities as that pattern resulted in the largest effect in reducing motion smear. Motion smear patterns that appear to be “moving” may increase its efficacy and reduce habituation, as the frontal vision in birds may be more tuned for the detection of movement (Martin, 2011). In this paper, we test the efficacy of contrast painting one rotor blade black (Figure 1), as proposed by Hodos (2003), to reduce avian collisions. Our prediction was that the painting would increase the visibility of the blades, as this reduces the visual smearing effect once the blades are rotating, and that this would lower the collision risk, as suggested by Hodos (2003). We tested this prediction in situ at the Smøla wind‐power plant in Norway using a Before–After–Control–Impact (BACI) approach based on long‐term fatality searches. The Smøla wind‐power plant provided us with a suitable study system because of its relative large number of wind turbines, and the existence of long‐term data on turbine fatalities before treatment (Bevanger et al., 2010). The Smøla archipelago is a coastal area rich in birdlife, and it has been designated an Important Bird Area (IBA) by Birdlife International (BirdLife International, 2020).

## 2 MATERIAL AND METHODS

### 2.1 Study area

**Figure 2:**
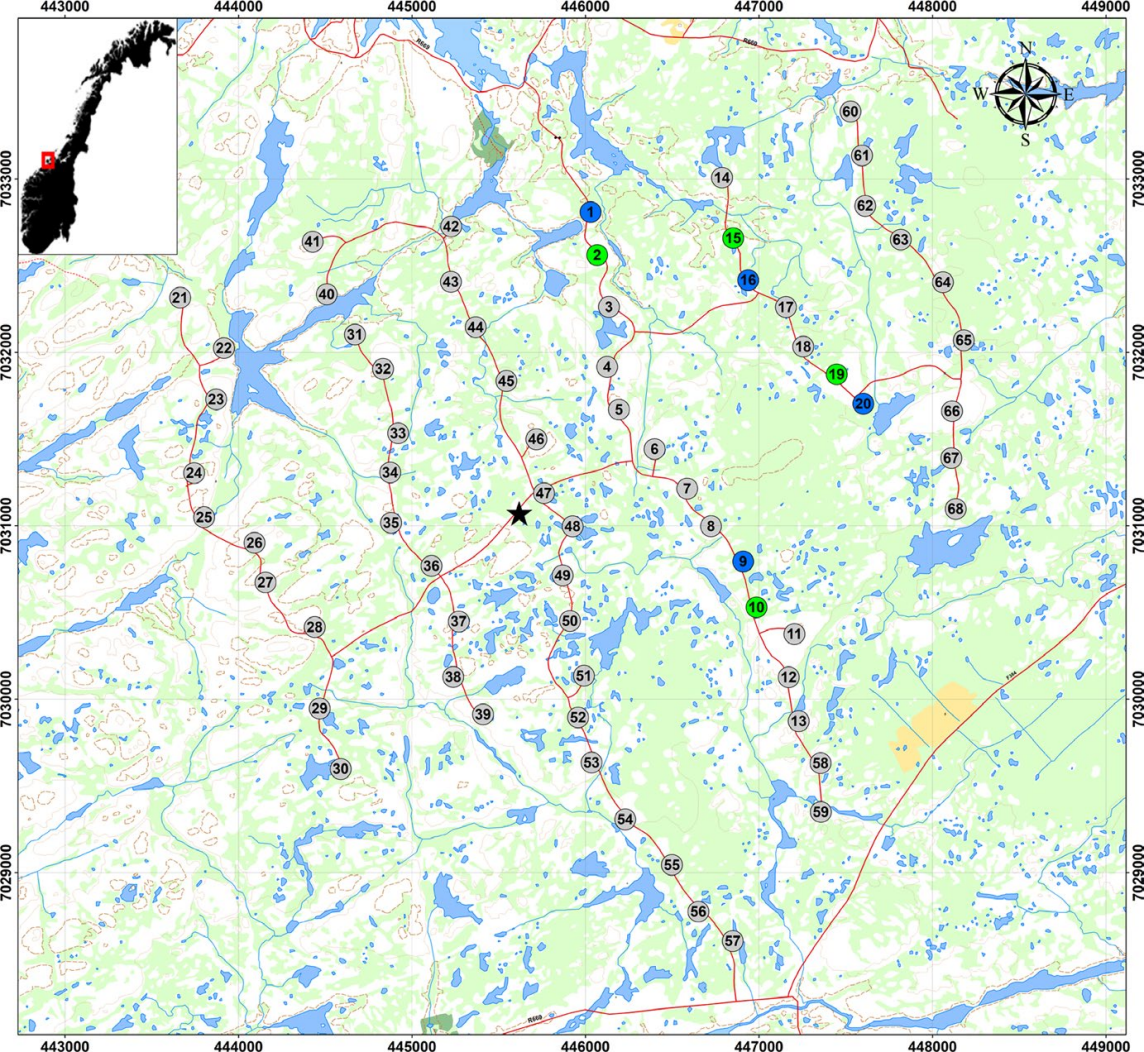
Set‐up of the experimental design at the Smøla wind‐power plant, Norway. Numbered circles indicate the wind turbines. Four turbines had painted rotor blades (blue) with adjacent control turbines (green)

Smøla is an archipelago located off the coast of Møre & Romsdal County, Central Norway (63°24′N, 8°00′E) (Figure 2) and consists of a large main island together with approximately 5,500 smaller islands, islets, and small skerries. The habitats are characterized by relatively flat open terrain consisting of heath and marsh vegetation, and rocky outcrops interspersed with minor bogs and lakes. The highest peak on the main island is only 69 m above sea level. The Smøla wind‐power plant is situated on the northwest side of the main island (May, Nygård, Dahl, & Bevanger, 2013). It was built in two phases by the Norwegian energy company Statkraft. The first phase consisting of 20 2.1 MW turbines was finished in September 2002, while the second phase with an additional 48 2.3 MW turbines became operational in August 2005. Since 2005, the wind‐power plant consists of 68 turbines (hub height: 70 m; rotor blade length 40 m). The wind‐power plant covers an area of 17.83 km^2^; represented by the minimum convex polygon (i.e., envelope) around the outermost turbines including a 200‐m buffer. The wind‐power plant area is accessible through unpaved maintenance roads. Between August 1 and August 8, 2013, one of three rotor blades were painted black at four of the 2.1 MW turbines with previously recorded carcasses (turbine IDs 1, 9, 16, and 20). Neighboring turbines, also with previously recorded carcasses, were defined as control turbines (turbine IDs 2, 10, 15, and 19) for fatality searches. The experiment was executed with all required permits from Statkraft, municipality of Smøla, Norwegian Energy and Water Resources Directorate (all painting experiment) and the Civil Aviation Authority (painting experiment and use of radar).

### 2.2 Fatality searches using trained dogs

The best way to assess the efficacy of mitigation measures is to follow a BACI approach; comparing painted with control turbines before and after treatment. Prior to the treatment, extensive and long‐term baseline data from beginning of 2006 and onwards were available from regular fatality searches using trained dogs (Bevanger et al., 2010; Nygård et al., 2010). Monitoring continued until the end of 2016, rendering seven and a half years before and three and a half years after treatment.

Searches for bird carcasses were performed at regular intervals in a radius of 100 m around the turbines, using dogs that were trained to find bird carcasses and feathers. Searches were performed by scanning the ca. 31,400 m^2^ area upwind on days without much precipitation to maximize the likelihood of the dogs acquiring scent of dead birds or feathers. The dogs were trained to lie down at the object when such targets were found. In addition, carcasses found by personnel and passers‐by were also recorded. There are no restraints on the public of entering the wind farm on foot or bicycle. Maintenance personnel were instructed by the wind‐farm owner, Statkraft, to secure and freeze dead bird found at the turbines and make them available to the researchers. Often the carcasses were found close to turbines or maintenance roads. The most common species found were willow ptarmigan (*Lagopus lagopus*), white‐tailed eagle (*Haliaeetus albicilla*), common snipe (*Gallinago gallinago*), hooded crow (*Corvus cornix*), and meadow pipit (*Anthus pratensis*) (Table S1). Eagles are large conspicuous birds and will therefore often catch the attention of passers‐by. Ptarmigan are often found near the turbine base as they are suspected to collide with the actual tower (see Stokke, Nygård, Falkdalen, Pedersen, & May, 2020). Mammalian scavengers do not exist on the island of Smøla, minimizing any removal bias. Search intensity has varied over time to address the research questions of consecutive research projects (see Figure S1). The overall background, methods, and findings during 2007–2010 are reported in Bevanger et al. (2010).

### 2.3 Statistical analyses

We tested for effects of rotor blade painting on annual fatality rates, before and after painting following a BACI approach (B = before, A = after; C = control, and I = impact). The analyses were performed by grouping recorded number of carcasses per turbine and by year (2006–2016). For each turbine, the number of recorded carcasses and the number of searches performed were summarized per year. In the analyses, the annual number of recorded carcasses was used as response variable while including the logarithm of the search effort as an offset term (rendering annual fatality rates per turbine). The annual fatality rates at the four control turbines were compared with the painted turbines (CI) before and after painting (BA). The interaction term (BA:CI) evaluates changes at the impact turbines before and after painting relative to control turbines. In the remainder of the text, we refer to this interaction effect. We chose to use neighboring turbines as controls to ensure similar spatial conditions for comparison. To control for any potential effects of turbine IDs and year, random effects were included using a generalized linear mixed‐effects model with a Poisson distribution using the glmer function of the lme4 library (Bates, Maechler, Bolker, & Walker, 2015) in the statistical software program R 3.3.3 (R Core Team, 2016). To control for potential overdispersion in the data, we also included an observation‐level random effect (Harrison, 2014). To test for the potential effect of birds potentially being forced toward neighboring turbines due to deterrence from painted turbines, we first verified whether these controls (as “pseudo‐impact” turbines) differed significantly in annual fatality rates from other untreated turbines (as unrelated controls) before–after treatment. Willow ptarmigan carcasses were excluded from the analyses as these are known to collide with the tower base (see Stokke et al., 2020).

To further assess the overall effects of painting on separate bird groups (raptors, passerines, and waterbirds), we estimated the (Poisson) probability for obtaining the actual recorded number of carcasses in the period after painting, assuming no treatment effect. We estimated the expected number of fatalities over the total number of searches executed at the painted turbines after painting with an expected mean annual fatality rate assuming no effect of the treatment: ${\text{rate}}_{\text{IA}}=\frac{{\text{rate}}_{\text{CA}}}{{\text{rate}}_{\text{CB}}}\cdot {\text{rate}}_{\text{IB}}$ using the rpois function where the rates indicate the four different groups of the BACI design. The probability was obtained through a simulation with 10,000 iterations to derive the proportion of times we obtained the same number of carcasses as were actually recorded. Also, the expected number of fatalities (mean and *SD*) assuming no treatment effect was calculated.

## 3 RESULTS

Throughout the wind‐power plant, 9,557 turbine searches have been performed in the period 2006–2016, whereby 464 carcasses have been recorded (Table S1). At the eight study turbines combined, 1,275 individual turbine searches were performed, during which 82 carcasses were found (including 40 willow ptarmigan not included in the study; Table 1, Table S1). Only two of these carcasses were found opportunistically outside of the regular searches: one willow ptarmigan which was not included in the analyses and one white‐tailed eagle that would have been found at the next regular search.

**Table 1:** Total number carcasses recorded during the fatality searches at the turbines included in the experiment (2006–2016). Willow ptarmigan was excluded from the analyses as this species is known to collide with the turbine tower base (see Stokke et al., 2020)

Species name	Latin name	Before		After	
		Control	Impact	Control	Impact
White‐tailed eagle	*Haliaeetus albicilla*	1	6	0	0
Common kestrel	*Falco tinnunculus*	0	0	2	0
Greylag goose	*Anser anser*	0	0	1	1
Northern shoveler	*Anas clypeata*	0	1	0	0
Eurasian teal	*Anas crecca*	0	1	0	0
Common snipe	*Gallinago gallinago*	2	0	4	0
European golden plover	*Pluvialis apricaria*	1	1	0	0
Wader spp.	*Charadriiformes*	0	0	1	0
Gull spp.	*Larinae*	0	1	0	0
Common raven	*Corvus corax*	1	0	0	0
Hooded crow	*Corvus cornix*	0	1	0	3
Parrot crossbill	*Loxia pytyopsittacus*	1	0	0	0
Red crossbill	*Loxia curvirostra*	0	0	1	0
European greenfinch	*Chloris chloris*	0	0	1	0
Meadow pipit	*Anthus pratensis*	1	0	3	0
Common blackbird	*Turdus merula*	0	0	1	0
Thrush spp.	*Turdus*	0	0	2	0
Passerine spp.	*Passeriformes*	0	0	0	1
Willow ptarmigan	*Lagopus lagopus*	8	5	15	12
Bird spp.	*Aves*	0	0	2	1
Total, excl. Willow ptarmigan		7	11	18	6
Number of fatality searches		345	351	289	290

**Figure 3:**
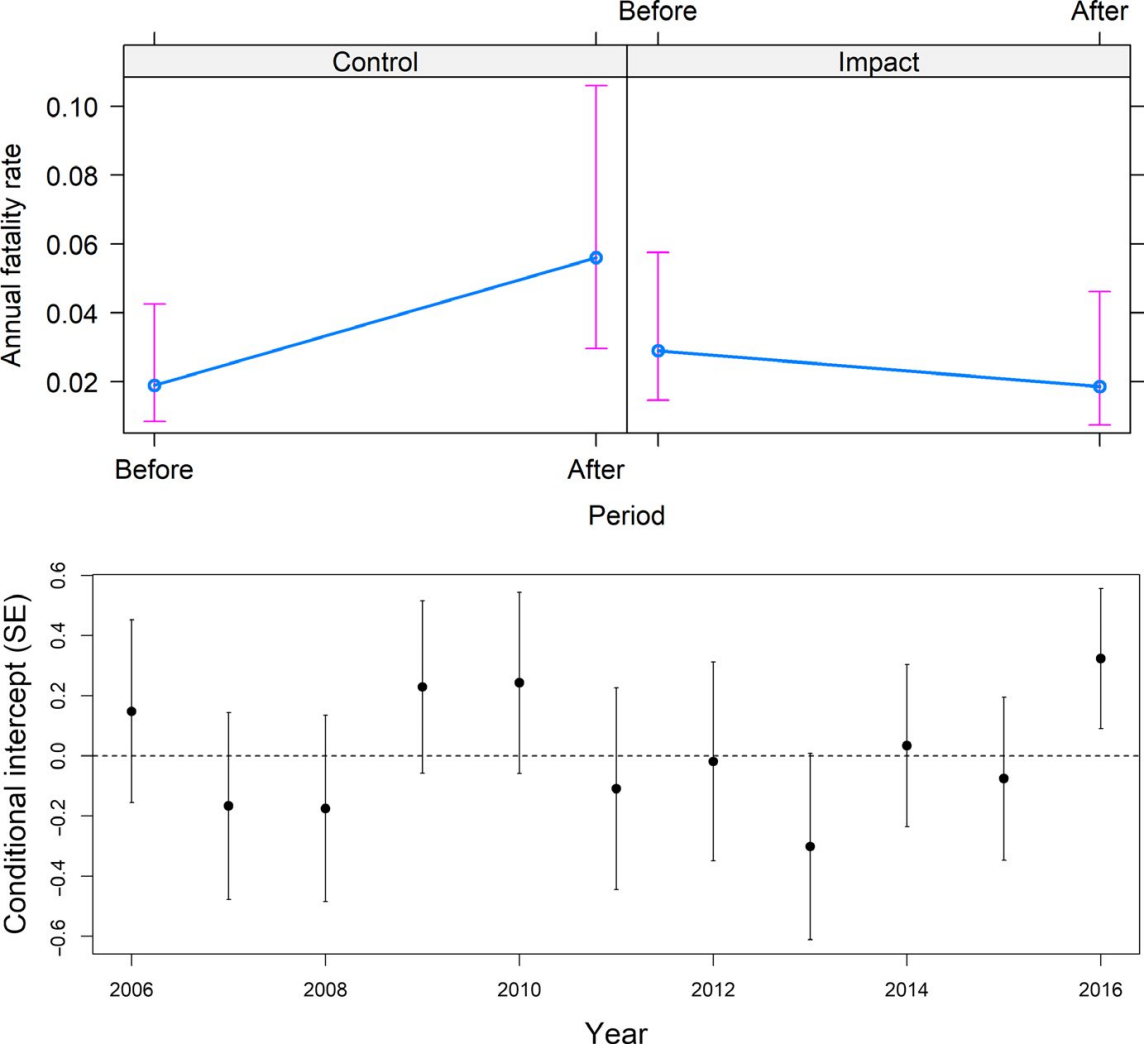
Result of the BACI‐model testing for the effect of contrast painting of rotor blades on the annual fatality rates at the Smøla wind‐power plant (upper panel). The lower panel shows the variation in annual fatality rates over the years

**Figure 4:**
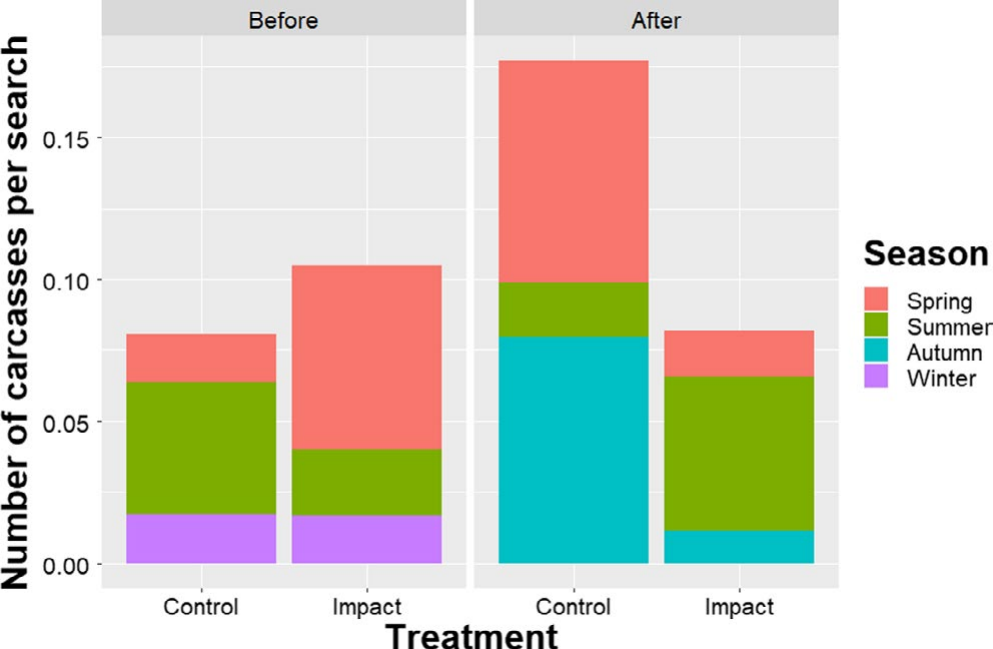
Seasonal number of recorded carcasses per search before and after painting at the eight experimental turbines (control and impact) at Smøla wind‐power plant in the period 2006–2016. Winter: 15.12–14.03; Spring: 15.03–14.06; Summer: 15.06–14.09; and Autumn: 15.09–14.12

While the number of recorded carcasses increased at the control turbines (7 vs. 18), they decreased at the treated turbines (11 vs. 6 [expected: 28]) (Table 1). We found no effect of birds having a higher probability of collision at the neighboring control turbines due to painting. This was tested by comparing annual fatality rates at control turbines to other untreated turbines before–after treatment within the wind‐power plant (*z* = −0.033, *p* = .974). The BACI model indicated that the annual fatality rate was significantly reduced after painting at the painted turbines (*z* = −2.279, *p* = .023, *n* = 96; Table 2). Overall, there was an average 71.9% reduction in the annual fatality rate after painting at the painted turbines relative to the control turbines (95% CI: 61.8%–79.1%;Figure 3 upper panel). However, the annual fatality rates fluctuated considerably between years (Figure 3 lower panel), stressing the necessity of a long‐term study. Seasonally, fatality rates (across years) were strongly reduced at the painted turbines after treatment during spring and autumn, but increased during summer (Figure 4). When grouping data by season instead of years, painting reduced seasonal fatality rates by 70.9% (95% CI: 61.7%–77.7%; *z* = −2.003, *p* = .042, *n* = 64). The variation among years was more pronounced than seasonal variation (variance Year: 0.125; variance Season: 0.005).

**Table 2:** Model estimates testing the effect of painting on the annual fatality rate of birds found at the Smøla wind‐power plant (2006–2016) using a Before–After–Control–Impact (BACI) design. The model controlled for (log‐transformed) search effort using an offset term

Fixed effects	Annual fatality rates				Seasonal fatality rates			
	Estimate	*SE*	*z*‐Value	*p*	Estimate	*SE*	*z*‐Value	*p*
Intercept	−3.966	0.414	−9.588	<.001	−4.020	0.443	−9.070	<.001
BA – After	1.085	0.501	2.165	.030	1.073	0.500	2.144	.032
CI – Impact	0.426	0.481	0.886	.376	0.457	0.536	0.852	.394
BA:CI	−1.529	0.671	−2.279	.023	−1.512	0.744	−2.033	.042

Random intercepts	Variance	*SD*	*N*		Variance	*SD*	*N*	
Record	7.391 × 10^–10^	2.719 × 10^–5^	96		0.2524	0.502	64	
Turbine	0.000	0.000	8		1.326 × 10^–10^	1.152 × 10^–5^	8	
Season					4.796 × 10^–3^	6.926 × 10^–2^	4	
Year	0.125	0.353	11					

The (Poisson) probability for recording the actually found four passerine carcasses in the period after painting, given a prior mean annual fatality rate of 0.022, was estimated to 0.127. All else being equal, the expected number of passerine fatalities, assuming no effect of the treatment, would have been 6.2 ± 2.5 *SD* over the entire period after painting. The probability for recording one water bird carcasses in the period after painting was 0.200 (prior mean annual fatality rate: 0.009, expected number of fatalities: 2.5 ± 1.6 *SD*). The probability of recording no raptor carcasses in the period after painting was <0.001 (prior mean annual fatality rate: 0.035, expected number of fatalities 10.2 ± 3.2 *SD*). White‐tailed eagles have been the primary species of concern at the Smøla wind‐power plant. Before the experiment, six white‐tailed eagle carcasses were found dead at painted turbines (351 searches) but none at control turbines. Afterward, no carcasses were recorded at neither treated nor control turbines (290 searches) (Table 1). Therefore, separate tests were performed for treated and control turbines, assuming equal fatality rates before and after painting. For white‐tailed eagles alone, the likelihood of recording no fatalities after treatment, all else being equal, was 0.009 at the painted turbines (pretreatment rate: 0.016, expected: 4.6 ± 2.1 *SD*) while being 0.491 at the control turbines (pretreatment rate: 0.002, expected: 0.7 ± 0.8 *SD*).

## 4 DISCUSSION

This study presents the outcomes of the first dedicated in situ experiment extensively testing the efficacy of passive markings to reduce collision mortality at wind turbines (but see Stokke et al., 2020). The annual fatality rate was significantly reduced at the treated turbines by over 70%. The in situ experiment was performed comparing only four treated turbines to the neighboring four untreated turbines. We must therefore be careful what we deduce from the experiment given the limited number of turbine pairs. However, the experiment ran over a long timeframe, encompassing seven and a half years pretreatment and three and a half years post‐treatment (i.e., time‐for‐space substitution). Despite the spatial limitation, the long‐term study does account for annual variability, and given the lack of a clear temporal pattern across years (Figure 3 lower panel), we saw no clear evidence for habituation effects. Over time, birds may build up a cognitive spatial map of its surroundings where wind turbines may function as landmarks (cf. May et al., 2015). Familiarity with the turbines through associative learning may make them less sensitive to any temporal effects, leading to habituation and increased risk of collision. The painted rotor blades may, however, also have made birds more aware (or vigilant) of those turbine locations, leading to anticipatory evasion (May et al., 2015). We did not find any indications for spatial effects where birds were being “forced into” neighboring turbines due to an evasive response to the painted rotor blades. However, the possibility of such effects should be tested through monitoring the long‐term efficacy of potential future implementations of rotor painting at other sites. The reason why the fatality rates increased, while taking into account annual variation and search effort, is uncertain. The fatality searches were executed by different teams (personnel and dogs) before versus after treatment, however, according to the same protocol. We cannot rule out general changes in the local abundance of birds, although we do not have data on this. Still, the BACI design is robust for such effects, and the outcome is unaffected by such possibilities as we are solely interested in the interaction term between treatment and period (Loss, Will, & Marra, 2012).

The painting regime especially reduced raptor fatalities. Bird fatalities within the entire wind‐power plant included a suite of species with varying susceptibility to collision with the wind turbines. Of these species, willow ptarmigan (excluded from this study as they were expected to collide with the turbine tower, see Stokke et al., 2020) and white‐tailed eagle had the highest number of recorded carcasses. Before the experiment, six white‐tailed eagles were found dead at to‐be‐painted turbines but after painting none. The reduction of these carcasses was assessed to be very unlikely due to random chance. Norway is considered a stronghold for this species, and the Smøla archipelago supports very high densities (Dahl, Bevanger, Nygård, Røskaft, & Stokke, 2012). This in part also explains their susceptibility to wind turbines. Earlier studies indicated that while not clearly adjusting their flight behavior in the vicinity of the wind turbines (Dahl et al., 2013), they were partially displaced from the wind‐power plant footprint (May et al., 2013). This in turn affected their breeding success within the wind‐power plant footprint (Dahl et al., 2012), their collision risk (May et al., 2010; May, Nygård, Dahl, Reitan, & Bevanger, 2011), and ultimately locally affected population growth rates within 1 km of the wind‐power plant (Dahl, 2014). Still, there are no indications that white‐tailed eagle densities have declined on the archipelago. The number of recorded carcasses throughout the wind‐power plant has also remained stable up to and including 2019 (T. Nygård unpublished data). The current population status will be re‐evaluated in the coming years. Raptors in general are known to be especially susceptible to collisions with wind turbines (Thaxter et al., 2017). This has often been explained by their soaring flight, displaying and foraging behavior at rotor‐swept height (Dahl et al., 2013). However, also other species including the meadow pipit and common snipe are known to have aerial displaying behavior which could make them vulnerable to collision. Hooded crows are common birds that are known to scavenge on wind turbine fatalities (Bevanger et al., 2010).

The painting experiment had most effect for raptors. This may well be explained by the higher visual acuity of (especially larger) raptors enabling sharp sight at larger distances (Bringmann, 2019; Fernandez‐Juricic, Erichsen, & Kacelnik, 2004). This ability may well enable raptors to be better capable to discern the painted rotor blades when approaching. White‐tailed eagles may in addition also be expected to have a wider visual field compared to other raptor species, as this was found to be the case for the taxonomically related bald eagle (*Haliaeetus leucocephalus*) (Potier et al., 2018). Their visual field was found to be similar to those of vultures due to their aquatic foraging strategy (Potier et al., 2018). The visual acuity of diurnal birds of prey for stationary objects, but not for rapidly changing objects, strongly decreases in dim light (Bringmann, 2019; Mitkus, Potier, Martin, Duriez, & Kelber, 2018). Whether this may limit the efficacy of the painting regime under dim light conditions remains as yet unclear. In such situations, blade conspicuity may for diurnal birds be enhanced by potentially including stronger temporal cues using varying blade patterns (e.g., reflective paint, myriad reflectors, and flickering lights) (Hodos, 2003). The visual acuity of birds, relating both to the spatial (Gaffney & Hodos, 2003) and temporal resolution (Bostrom et al., 2016), may well enable birds to anticipate the turbines with reduced motion smear more rapidly (cf. anticipatory evasion, May, 2015). The ultrarapid vision of birds possibly enables them to quickly become aware of the turbines with a painted rotor blade (cf. Bostrom et al., 2016). Although ultraviolet paint (or other coloring) or lighting has also been proposed, these have so far shown limited efficacy (Hodos, 2003; May, Åström, Hamre, & Dahl, 2017; Young, Erickson, Strickland, Good, & Sernka, 2003). In addition, not all species are sensitive within the ultraviolet spectrum (Lind, Mitkus, Olsson, & Kelber, 2014). The advantage of using passive visual marking over lighting is that the former will be much easier to implement. A similar advantage can be expected relative to operational measures, such as shutdown on command or feathering, as it does not require ancillary detection technology (May et al., 2015).

Painting the rotor blades was demanding given that they had to be painted while in‐place. This meant using a lift which was attached to the hub and paint downwards therefrom. Therefore, the topmost section closest to the hub (ca. 12 m) could not be painted. The work had to be done in calm weather, using specialized personnel (rappelling experience). However, when implemented before construction, the cost would be minimized. Also, visual observation by technical staff at the wind‐power plant confirmed that no detrimental effects could be observed concerning paint quality over several years of usage (Birger Træthaug, pers. comm.). No negative reactions from local human inhabitants are known to us. For this study, we maximized the contrast by applying black paint. What remains to be tested is whether other color regimes will be equally effective, for example, red stripes as used for aviation warning purposes, green paint to reduce visibility (to humans) in the landscape or optical or holographic coatings. In the experiments by Hodos (2003), yellow and red, but especially green blades had slight—but nonsignificant—visibility advantages over black; which was therefore suggested be simplest and most effective to implement.

## 5 CONCLUSION

Applying contrast painting to the rotor blades resulted in significantly reduced the annual fatality rate (>70%) for a range of birds at the Smøla wind‐power plant. We recommend to either replicate this study, preferably with more treated turbines, or to implement the measure at new sites and monitor collision fatalities to verify whether similar results are obtained elsewhere, to determine to which extent the effect is generalizable. It is of the utmost importance to gain more insights into the expected efficacy of promising mitigation measures through targeted experiments and learning by doing, to successfully mitigate impacts on birdlife and to support a sustainable development of wind energy worldwide.

## CONFLICT OF INTEREST

None declared.

## AUTHOR CONTRIBUTIONS

**Roel May:** Conceptualization (lead); Formal analysis (lead); Funding acquisition (lead); Methodology (lead); Project administration (lead); Visualization (lead); Writing‐original draft (equal); Writing‐review & editing (lead). **Torgeir Nygard:** Conceptualization (supporting); Data curation (supporting); Formal analysis (supporting); Project administration (supporting); Writing‐original draft (equal); Writing‐review & editing (equal). **Ulla Falkdalen:** Data curation (equal); Writing‐original draft (supporting). **Jens Åström:** Formal analysis (supporting); Writing‐original draft (supporting); Writing‐review & editing (supporting). **Øyvind Hamre:** Data curation (equal); Writing‐original draft (supporting); Writing‐review & editing (supporting). **Bård Gunnar Stokke:** Data curation (supporting); Project administration (supporting); Writing‐original draft (supporting); Writing‐review & editing (supporting).

## Supporting information

Fig. S1

Table S1

## Data Availability

Table S1 and Figure S1 as well as the R scripts and data files to perform the analyses are supplied as supplementary materials at the Mendeley Data repository (http://dx.doi.org/10.17632/dc7kg5b8vd.1).
