# Supplementary figures and images for: Paint it black: Efficacy of increased wind turbine rotor blade visibility to reduce avian fatalities
Source: Ecol Evol. 2020 Jul 26;10(16):8927–35. doi: 10.1002/ece3.6592 (PMC7452767; doi:10.1002/ece3.6592)

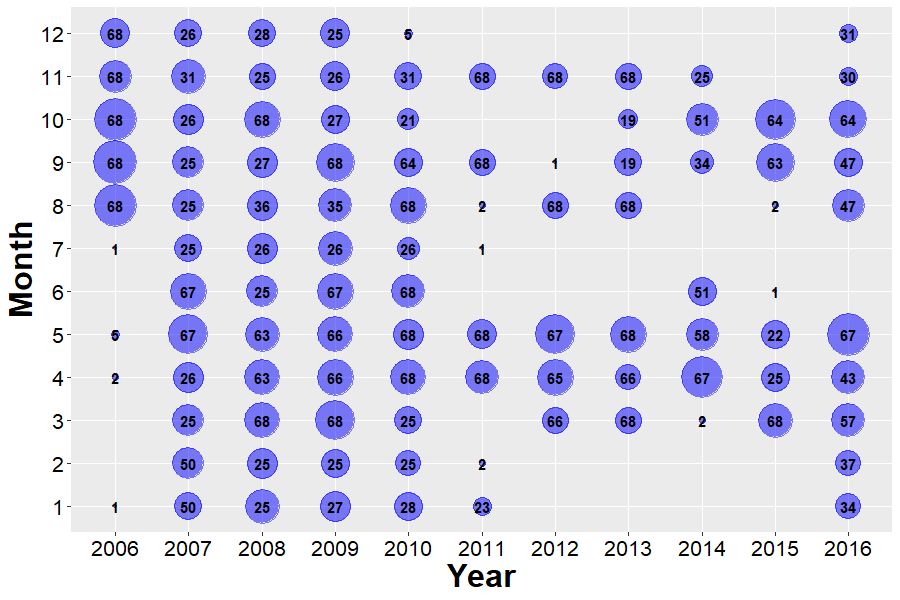

Supplement: Supplementary file 1 — Fig. S1 [file ECE3-10-8927-s001.tiff]
