# Supplementary material for: Paint it black: Efficacy of increased wind turbine rotor blade visibility to reduce avian fatalities
Source: Ecol Evol. 2020 Jul 26;10(16):8927–35. doi: 10.1002/ece3.6592 (PMC7452767; doi:10.1002/ece3.6592)

**SUPPLEMENTARY INFORMATION**

**Paint it black: Efficacy of increased wind-turbine rotor blade visibility to reduce avian fatalities**

Roel May^1,*^, Torgeir Nygård^1^, Ulla Falkdalen^2^, Jens Åström ^1^, Øyvind Hamre^1^, Bård G. Stokke^1^

^1^ *Norwegian Institute for Nature Research, P.O. Box 5685 Torgarden, 7485 Trondheim, Norway*

^2^ *Frösö-Berge 110, SE-832 96 Frösön, Sweden*

^*^ Email: [roel.may@nina.no](mailto:roel.may@nina.no), Phone: +47 95785995, ORCID: <https://orcid.org/0000-0002-6580-4064>

**TABLE S1** Total number of carcasses recorded near all turbines at the Smøla wind-power plant (n = 464) during fatality searches (2006-2016). The number found at the experimental turbines (both treatment and control) is indicated in brackets (n = 42).

| **Group** | **Common name** | **Scientific name** | **N** |
| --- | --- | --- | --- |
| Accipitriformes | Golden eagle | *Aquila chrysaetos* | 2 (0) |
| Accipitriformes | Merlin | *Falco columbarius* | 1 (0) |
| Accipitriformes | Gyrfalcon | *Falco rusticolus* | 1 (0) |
| Accipitriformes | Common kestrel | *Falco tinnunculus* | 4 (2) |
| Accipitriformes | White-tailed eagle | *Haliaeetus albicilla* | 71 (7) |
| Anseriformes | Northern shoveler | *Anas clypeata* | 1 (1) |
| Anseriformes | Eurasian teal | *Anas crecca* | 3 (1) |
| Anseriformes | Mallard | *Anas platyrhynchos* | 6 (0) |
| Anseriformes | Greylag goose | *Anser anser* | 7 (2) |
| Anseriformes | Duck spp. | *Anseriformes* | 2 (0) |
| Anseriformes | Whooper swan | *Cygnus cygnus* | 2 (0) |
| Anseriformes | Common merganser | *Mergus merganser* | 1 (0) |
| Anseriformes | Red-breasted merganser | *Mergus serrator* | 2 (0) |
| Charadriiformes | Little auk | *Alle alle* | 1 (0) |
| Charadriiformes | Dunlin | *Calidris alpina* | 1 (0) |
| Charadriiformes | Wader spp. | *Charadriiformes* | 5 (1) |
| Charadriiformes | Northern fulmar | *Fulmarus glacialis* | 1 (0) |
| Charadriiformes | Common snipe | *Gallinago gallinago* | 32 (6) |
| Charadriiformes | Eurasian oystercatcher | *Haematopus ostralegus* | 3 (0) |
| Charadriiformes | Gull spp. | *Larinae* | 4 (1) |
| Charadriiformes | European herring gull | *Larus argentatus* | 4 (0) |
| Charadriiformes | Common gull | *Larus canus* | 1 (0) |
| Charadriiformes | Great black-backed gull | *Larus marinus* | 1 (0) |
| Charadriiformes | European golden plover | *Pluvialis apricaria* | 12 (2) |
| Charadriiformes | Black-legged kittiwake | *Rissa tridactyla* | 1 (0) |
| Charadriiformes | Common redshank | *Tringa totanus* | 1 (0) |
| Passeriformes | Common redpoll | *Acanthis flammea* | 1 (0) |
| Passeriformes | Meadow pipit | *Anthus pratensis* | 19 (4) |
| Passeriformes | European greenfinch | *Chloris chloris* | 1 (1) |
| Passeriformes | Common wood pigeon | *Columba palumbus* | 2 (0) |
| Passeriformes | Common raven | *Corvus corax* | 2 (1) |
| Passeriformes | Hooded crow | *Corvus cornix* | 24 (4) |
| Passeriformes | Barn swallow | *Hirundo rustica* | 1 (0) |
| Passeriformes | Twite | *Linaria flavirostris* | 1 (0) |
| Passeriformes | Red crossbill | *Loxia curvirostra* | 1 (1) |
| Passeriformes | Parrot crossbill | *Loxia pytyopsittacus* | 1 (1) |
| Passeriformes | Crossbill spp. | *Loxia spp.* | 1 (0) |
| Passeriformes | Northern wheatear | *Oenanthe oenanthe* | 7 (0) |
| Passeriformes | Passerine spp. | *Passeriformes* | 10 (1) |
| Passeriformes | Common starling | *Sturnus vulgaris* | 5 (0) |
| Passeriformes | Redwing | *Turdus iliacus* | 1 (0) |
| Passeriformes | Common blackbird | *Turdus merula* | 1 (1) |
| Passeriformes | Fieldfare | *Turdus pilaris* | 2 (0) |
| Passeriformes | Thrush spp. | *Turdus spp.* | 3 (2) |
| Other species | Grey heron | *Ardea cinerea* | 4 (0) |
| Other species | Bird spp. | *Aves* | 14 (3) |
| Other species | Northern bat | *Eptesicus nilssonii* | 1 (0) |
| Other species | Willow ptarmigan | *Lagopus lagopus* | 192 (40) |

**FIGURE S1** Search effort for wind-turbine fatalities at the Smøla wind-power plant, Norway. The size of the bubbles indicates the total number of turbines searched during each month and year. The label numbers indicate the number of unique turbines searched during that month.


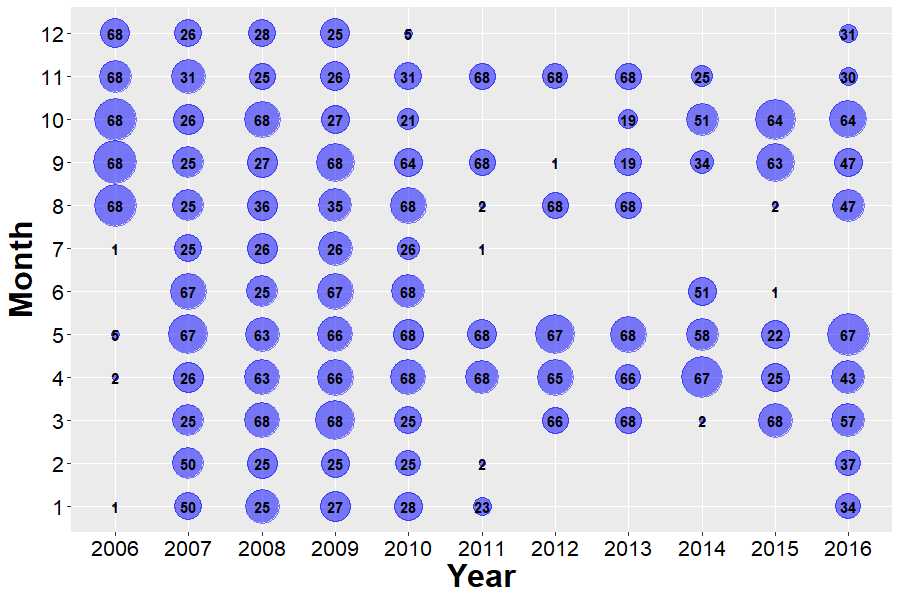

Supplement: Supplementary file 2 — Table S1 [file ECE3-10-8927-s002.docx]
